# Global measurement of intimate partner violence to monitor Sustainable Development Goal 5

**DOI:** 10.1186/s12889-022-12822-9

**Published:** 2022-03-08

**Authors:** Kathryn M. Yount, Yuk Fai Cheong, Zara Khan, Irina Bergenfeld, Nadine Kaslow, Cari Jo Clark

**Affiliations:** 1 Hubert Department of Global Health, Rollins School of Public Health, Emory University, 1518 Clifton Rd, NE, Room 7029, Atlanta, GA 30322 USA; 2 Department of Psychology, Emory University, 36 Eagle Row, Atlanta, GA 30322 USA; 3 University of Texas Southwestern Medical School, 5323 Harry Hines Blvd, Dallas, TX 75390 USA; 4 Department of Psychiatry and Behavioral Sciences, Emory University School of Medicine, 12 Executive Park Dr, Atlanta, GA 30329 USA

**Keywords:** Alignment optimization, Controlling behaviours, Cross-national, Measurement invariance testing, Physical intimate partner violence, Sustainable development goal 5

## Abstract

**Background:**

One third of women experience intimate partner violence (IPV) and potential sequelae. Sustainable Development Goal (SDG) 5.2—to eliminate violence against women, including IPV—compels states to monitor such violence. We conducted the first global measurement-invariance assessment of standardised item sets for IPV.

**Methods:**

Demographic and Health Surveys (DHS) from 36 Lower−/Middle-Income Countries (LMICs) administering 18 IPV items during 2012–2018 were included. Analyses were performed separately for two items sets: lifetime physical IPV (seven items) and controlling behaviours (five items). We performed country-specific exploratory and confirmatory factor analyses (EFA/CFA). Datasets meeting benchmarks for acceptable item loadings and model-fit statistics were included in multiple-group CFA (MGCFA) to test for *exact measurement invariance*. Based on findings, alignment optimization (AO) was performed to assess *approximate measurement invariance* (< 25% of model parameters non-invariant). For each item set, national rankings based on AO-derived scores and on prevalence estimates were compared. AO-derived scores were correlated with type-specific IPV prevalences to assess correspondence.

**Results:**

National rates of physical IPV (5.6–50.5%) and controlling behavior (25.9–84.7%) varied. For each item set, item loadings and model-fit statistics were adequate in country-specific, unidimensional EFAs and CFAs. Both unidimensional constructs lacked exact invariance in MGCFA but achieved approximate invariance in AO analysis (12.3% of model parameters for physical IPV and 6.7% for controlling behaviour non-invariant). For both item sets, national rankings based on AO-derived scores were distributed similarly to rankings based on prevalence. However, estimates often were not significantly different cross-nationally, precluding national-level comparisons regardless of estimation strategy. Three physical-IPV items (slap, twist, choke) and two controlling-behaviour items (meet female friends; contact with family) warrant cognitive testing to improve their psychometric properties. Correlations of AO-derived scores for physical IPV (0.48–0.66) and controlling behaviours (0.49–0.87) with prevalences of lifetime physical, sexual, psychological IPV as well as controlling behaviour varied.

**Conclusions:**

Seven DHS lifetime physical-IPV items and five DHS controlling-behaviour items were approximately invariant across 36 LMICs spanning five world regions, such that cross-national comparisons of factor means are reasonable. Measurement-invariance testing over time will inform their utility to monitor SDG5.2.1; cross-national, cross-time measurement-invariance testing of improved sexual and psychological IPV item-sets is needed.

**Supplementary Information:**

The online version contains supplementary material available at 10.1186/s12889-022-12822-9.

## Introduction

*Intimate partner violence* (IPV)—or psychological, physical, and sexual violence and controlling behaviour perpetrated by a spouse or dating partner—is a global public-health problem. Approximately 27% (95% Confidence Interval [CI] 23–31%) of ever-partnered women 15–49 years have ever experienced physical and/or sexual IPV, with regional estimates ranging from 18 to 35% [1]. Adverse effects of IPV on women may include economic insecurity and physical-, mental-, behavioural-, sexual-, or reproductive-health conditions [2–9]. IPV compromises national economic development, costing an estimated 5% of world gross domestic product (GDP) and nearly 15% of GDP in Sub-Saharan Africa [10].

Given the health, social, and economic costs of IPV, United Nations’ bodies, treaties, and declarations have called for better statistics on the nature, prevalence, causes, and consequences of violence against women as a basis for its elimination [11]. This pressure led, in 2015, to Sustainable Development Goal (SDG) 5.2, which urges governments to “eliminate all forms of violence against all women and girls in public and private...” [12]. Widespread endorsement of SDG5.2 compels national governments to measure and to report rates of violence against women, including IPV (SDG5.2.1).

The decades leading up to SDG5.2 saw marked growth in the number of IPV prevalence surveys. These surveys relied on diverse scales and data-collection approaches [13], from small-scale, localised research, to large multi-country studies [14, 15], and ongoing surveillance of IPV in multipurpose national surveys. No gold standard exists for data collection on IPV, but the Centers for Disease Control and Prevention [16], World Health Organization (WHO) [17], and Demographic and Health Surveys (DHS) [18] have agreed best practices. These practices include direct inquiry about acts experienced within a clear timeframe; the use of multiple, behaviourally-specific questions to capture reported experiences of specific acts of IPV; reliance on appropriately trained interviewers; and support for respondents and interviewers [11].

The DHS domestic violence module (DVM) is the most commonly administered module that follows these best practices to measure IPV at the national level in lower- and middle-income countries (LMICs). The DHS is a flagship project of the United States Agency for International Development (USAID), which has invested several hundred million dollars in data collection since 1984 [19] and is a critical source of population and health data for LMICs [20]. The DHS DVM is optional; however, by the end of 2020, 65 countries had administered it at least once, and 39 countries had administered it more than once (range: 1–9 times) [18], documenting large differences in national IPV prevalence [1].

While the DHS is used to inform policies, prevention efforts, and response interventions, the DHS DVM has not undergone a rigorous psychometric assessment. It, therefore, is unknown whether questions in the module are measurement invariant across countries on a global scale, a critical precondition for national comparisons. Research by members of this team on DHS questions about the acceptability of IPV showed modest non-comparability of prevalence estimates across countries due to module-design factors, such as slight differences across surveys in the number, wordings, and introductory framing of the questions [21]. If not identified and accounted for, areas of non-comparability may distort estimated differences in national IPV prevalence [21], with potential implications for national policies and the allocation of resources for prevention and response [22]. Addressing this knowledge gap now is critical, since the number of countries monitoring IPV will only increase with SDG5.2.

The objective of this paper is to perform the first comprehensive, global psychometric assessment of items developed to measure IPV in the DHS DVM. Using 36 national surveys conducted in LMICs during 2012–2018, we focused our main analysis on the item sets designed to measure lifetime physical IPV (seven items) and controlling behaviors (five items). The larger numbers of items in both sets made them more likely to be content valid, and violence researchers consider the physical IPV items to be more behaviourally specific and reliable than the psychological or sexual IPV items [23]. Our use of data from the DHS—the most geographically diverse source for nationally-representative data on IPV using similarly worded questions—enables us to make evidenced-based recommendations that are global in scope across LMICs. Our findings inform next steps in a global research agenda to improve measures of IPV to monitor SDG5.2.1.

## Methods

### Eligibility and sample

The DHS are multipurpose surveys administered to large, nationally-representative samples of households and randomly selected women of reproductive age (typically 15–49 years) in interviewed households. The DHS routinely collect data on women’s and children’s health. They use internationally recognised guidelines for survey methodology and for the ethical collection of data, including data on violence against women and girls (VAWG) [15, 24].

Eligible countries had completed a DHS between 2012 and 2018 (inclusive) and had administered the same 18 items measuring physical, sexual, or psychological IPV and controlling behaviours. Based on these criteria, the sample for this analysis included 36 DHS conducted in 36 LMICs (according to the World Bank classification system) and spanning five world regions.

Included DHS represented countries in Sub-Saharan Africa (22 countries), followed by countries in South and Southeast Asia (nine countries), Central Asia (two countries), North Africa/West Asia (two countries), and finally Latin America and the Caribbean (one country) (Table 1). Although a select sample, included DHS were conducted in demographically diverse national populations. For example, countries in the sample ranged widely in population size, from 516,000 people in the Maldives in its survey year to 1.35 billion in India in its survey year. Countries in the sample also ranged widely on the GINI index of income inequality retrieved for 2009–2018, from lower income inequality in the Kyrgyz Republic (GINI = 27.4) to higher inequality in Namibia (GINI = 59.1). Countries also ranged in gross national income per capita for 2018, from USD280 in Burundi to USD9310 in the Maldives, and in median grades of schooling completed for women of reproductive age in each DHS, from 3.0 in Nepal to 10.7 in the Philippines. Gender differences in the law, measured using the World Bank index on Women, Business, and the Law, ranged from 28.8 for Afghanistan to 86.9 for Zimbabwe, with higher scores indicating greater gender parity under the law (Supplemental Table S1). Finally, basic survey conditions varied somewhat across included DHS. The average survey team size ranged from 3 to 10 members. The number of training days ranged from 19 to 42, and the average interview duration ranged from 20 to 90 min, with a majority of DHS reporting an average duration of 30–60 min.

**Table 1:** Characteristics of included countries and Demographic and Health Surveys, *N* = 36 surveys across 36 countries 2012–2018

	Survey Characteristics				Demographic, Economic Conditions				Extent of Gender Equity in Laws on …^**f**^								
Country	Year	Team size, M^a^	Training Days	Interview, Min	Pop size^b^ (000’s)	GNI per capita^c^ (2018)	GINI^d^	Grades^e^ M WRA	Mobility	Work-place	Pay	Marriage	Parenthood	Entrepreneurship	Assets	Pension	WBL Index (2016)
**Central Asia**																	
Kyrgyz Republic	2012	7	21	30–60	6316	1220	27.4	5.0	100	100	25	100	40	100	100	50	76.9
Tajikistan	2017	6	28	30–60	9101	1010	34.0	5.1	100	50	25	100	80	100	100	50	75.6
**Latin America, Caribbean**																	
Haiti	2016–17	8	35	45	11,123	800	41.1	3.7	50	50	100	40	20	75	80	75	61.3
**N Africa, W Asia, Europe**																	
Armenia	2015–16	8	21	30–60	2952	3607	32.4	6.6	100	50	75	80	60	75	100	100	80.0
Egypt	2014	7–8	35	30–60	98,424	3380	31.8	4.9	50	75	0	0	20	75	40	100	45.0
**S, SE Asia**																	
Afghanistan	2015	8	23	30–60	37,172	550	.	3.7	25	25	0	20	20	75	40	25	28.8
Cambodia	2014	5–6	26	20	16,250	1380	.	3.3	100	100	75	80	20	100	100	25	75.0
India	2015–16	7	20	40–60	1,352,617	2020	35.7	4.1	100	100	0	100	20	75	80	75	68.8
Maldives	2016–17	7–9	30	30–60	516	9310	31.3	3.6	100	100	75	60	40	75	40	75	70.6
Myanmar	2015–16	6–7	25	30–60	53,708	1310	38.1	3.5	75	25	50	80	60	75	80	25	58.8
Nepal	2016	5	21	60	28,088	960	32.8	3.0	100	75	50	80	0	75	40	25	55.6
Pakistan	2017–18	6	28	60–90	212,215	1580	33.5	3.9	75	75	25	60	0	50	40	50	46.9
Philippines	2017	3–4	40	30–60	106,652	3830	44.4	10.7	75	100	100	60	60	100	60	75	78.8
Timor-Leste	2016	6	28	30–60	1268	1820	28.7	4.2	100	75	75	80	40	75	100	75	77.5
**Sub-Saharan Africa**																	
Angola	2015–16	6	42	·	30,810	3370	51.3	3.4	100	50	50	100	40	75	100	25	67.5
Benin	2017–18	6	28	20–30	11,485	870	47.8	3.2	50	100	50	80	60	75	80	100	74.4
Burundi	2016–17	7	35	30–60	11,175	280	38.6	4.1	100	100	75	60	40	75	60	75	73.1
Chad	2014–15	6	30	30–60	15,478	670	43.3	3.7	75	25	50	40	60	50	60	100	57.5
Comoros	2012	6	23	30–60	832	1320	45.3	4.1	75	75	100	40	40	75	40	25	58.8
DRC	2013–14	6	32	30–60	84,068	490	42.1	3.6	75	50	25	20	60	0	60	50	42.5
Ethiopia	2016	8	34	30–60	109,225	790	35.0	5.4	100	100	25	80	20	75	100	75	71.9
Gabon	2012	6	33	45–60	2119	6800	38.0	3.4	50	25	25	20	80	50	60	100	51.3
Gambia	2013	6	32	30–60	2280	700	35.9	3.7	100	50	75	100	60	75	60	75	74.4
Kenya	2014	6	24	30–60	51,393	1620	40.8	5.0	100	100	100	80	40	50	80	75	78.1
Malawi	2015–16	8	19	30–60	18,143	360	44.7	4.3	50	100	100	100	20	75	100	100	80.6
Mali	2018	5–6	33	30–60	19,078	830	33.0	3.1	50	25	25	20	60	75	80	100	54.4
Mozambique	2011	6	42	30–45	29,496	440	54.8	5.8	100	100	50	80	60	75	100	50	76.9
Namibia	2013	7	26	30–60	2448	5250	59.1	3.4	75	100	100	100	40	75	100	100	86.3
Nigeria	2013	8	28	30–60	195,875	1960	43.0	4.6	50	75	50	100	0	75	80	75	63.1
Rwanda	2014–15	7	28	30–60	12,302	780	45.1	4.1	75	100	75	60	20	75	100	75	72.5
Sierra Leone	2013	6	28	30–60	7650	500	34.0	3.8	100	25	50	100	0	75	80	75	63.1
Tanzania	2015–16	7	32	45–60	56,318	1020	40.5	5.3	100	100	100	80	60	75	60	100	84.4
Togo	2013–14	6	36	30–60	7889	650	43.1	3.4	100	100	100	60	60	75	80	100	84.4
Uganda	2016	7	30	30–60	42,723	620	42.8	4.2	50	100	100	80	40	75	40	75	70.0
Zambia	2013–14	10	35	30–60	17,352	1430	57.1	3.9	50	50	75	80	20	75	80	75	63.1
Zimbabwe	2015	8	24	30–60	14,439	1790	44.3	3.8	100	100	75	80	40	100	100	100	86.9

*Abbreviations: DRC* Democratic Republic of Congo, *M* Mean, *WRA* Women of reproductive age (15–49), *WBL* Women, Business, and the Law index

^a^Average size of each of the data collection teams including a supervisor and enumerators

^b^Population estimates from World Bank, survey year

^c^Gross national income per capita from 2018 World Bank estimates

^d^Gini coefficients estimate income inequality. Estimates retrieved from World Bank (2009–2018)

^e^Grades of schooling completed. From the DHS

^f^The World Bank Women, Business and the Law (WBL) index measures the extent of gender equity in laws across eight domains

### Data on IPV

The IPV-related questions in the DHS DVM [18] originated from the Revised Conflict Tactics Scales [15], a standardised instrument designed to capture behaviourally based acts of IPV ranging in severity from jealousy or anger for talking to other mean, to pushing or shoving, to the threat or actual use of a weapon. The DHS DVM has evolved to resemble more closely the instrument used by the WHO [17]. Specifically, the module includes three items to assess acts of psychological IPV, seven items to assess acts of physical IPV, three items to assess acts of sexual IPV, and five items to assess acts of male controlling behaviour. The occurrence of IPV is measured as the woman’s self-report of experiencing each IPV item: 1) ever in the lifetime of her referent relationship, and if yes, 2) with a standardised frequency in the 12 months before interview. Women’s reported experience of five controlling behaviours is measured without a specific timeframe or frequency. All items assess IPV in relation to the woman’s most recent spouse or partner. Supplemental Table S2 provides standard item wordings in English for each IPV item. Initial data exploration suggested that fewer than 2% of women in any included DHS sample had missing data on any single IPV item, and only 0.02% of all women (*n* = 65) across all 36 DHS had missing data on all IPV items.

### Statistical analysis

We used Stata [25] for data processing and descriptive analyses and Mplus [26] for all other analyses. The main statistical analyses involved four major steps. As a first step, we conducted descriptive analyses to understand country-specific missingness and prevalence for each IPV item and item-specific prevalence ranges across included countries. As a second step, we performed 36 country-specific factor analyses to *explore* and then to *confirm* dimensionality of each IPV item set, the magnitudes of item loadings, and overall model fit. For each country, the exploratory factor analysis (EFA) model was considered adequate if: item loadings were 0.35 or greater; model fit statistics met recommended benchmarks (the root mean square error of approximation (RMSEA) was about 0.08 or lower, and the comparative fit index (CFI) and Tucker-Lewis index (TLI) were about 0.95 or higher); and the results fit with theory [27]. We then conducted country-specific confirmatory factor analyses (CFA), including countries that met the above-mentioned model-fit criteria in the EFA. We used the same criteria for the item loadings and model-fit statistics to assess the adequacy of the fit of all CFA models. The EFA and CFA used the means and variance-adjusted weighted least squares estimators, which were appropriate for dichotomous responses (1 = [ever] experienced, 0 = did not [ever] experience the IPV item). The approach used pairwise deletion to handle missing data [28].

As a third step, for national datasets that exhibited adequacy with respect to item loadings and model-fit statistics, we considered two approaches to assess the cross-national measurement invariance of the models confirmed in country-specific CFAs. Initially, we performed multiple-group CFA (MGCFA) to test for *exact measurement invariance*. When using this approach, small measurement differences are assumed to be exactly zero [29]. Following this approach, we tested sequentially for *configural invariance*, or equivalence of the factor structure across countries; then *metric invariance*, or equivalence of the factor loadings across countries; and then *scalar invariance*, or equivalence of the factor loadings and thresholds (or intercepts) across countries. *Configural invariance* implies that the dimensional structure of the latent IPV factor is equivalent across countries, although the item loadings and intercepts are free to vary across countries; whereas *configural non-invariance* implies that the latent IPV factor has a different dimensional structure across countries. *Metric invariance* implies that each IPV item contributes to the latent IPV construct to a similar degree across countries. Conversely, *metric non-invariance* implies that at least one IPV item is related differently to the latent IPV construct across countries. *Scalar invariance* implies that the factorial scores are comparable across countries. Conversely, *scalar non-invariance* may indicate potential measurement bias and suggests that larger forces, such as cultural norms, may influence systematically how different populations respond to IPV items in ways that are unrelated to the latent IPV construct. We used Maximum Likelihood estimation, which is appropriate for dichotomous responses and allowed us to test separately for metric and scalar invariance [30].

In the exact invariance-testing framework, evidence of metric or scalar non-invariance leaves three analytical options: (1) investigate the source of the non-invariance by sequentially releasing or adding loading or intercept constraints and retesting the models until partial measurement invariance is achieved, (2) omit IPV items with non-invariant loadings or intercepts and retest the sequential invariance models, or (3) assume that the IPV construct is noninvariant and discontinue exact invariance testing. Given the large number of countries and small number of IPV items per set, we did not consider options (1) or (2) to be advisable.

Instead, as a fourth step, based on findings from the MGCFA, we used alignment optimization (AO) to assess *approximate measurement invariance* of the IPV items across countries. According to users of AO methods, the restriction of equal model parameters required by MGCFA may be overly strict, especially when many groups or time points are involved in the comparison (e.g., Davidov et al. [31]). The approximate measurement invariance approach allows, instead, for differences in these model parameters across groups by finding an optimal model with the minimal amount of measurement non-invariance. In the first step of AO [32], MGCFA was used to confirm cross-national configural invariance of the IPV factor model. In the second step of AO, if configural invariance was achieved, the factor means and variances of all but the reference group, which were fixed to 0 and 1, were estimated to minimise the total amount of non-invariance across all parameters. The quality of the alignment result, then, was determined by the percentage of loading and intercept parameters that displayed non-invariance. As a guide, a limit of 25% of non-invariant parameters or less indicated trustworthy results [33]. For higher percentages, a Monte Carlo simulation is advised to assess the quality of the results [33]. Monte Carlo simulations are based on the correlation between the population factor means and the estimated alignment factor means, computed over groups and averaged over replications. Correlations of at least 0.98 produce reliable factor means [33]. Like MGCFA, AO employed maximum likelihood estimation, which used all available data, assuming data were missing at random [28, 33].

At the initial stages of analysis, we attempted to follow the above steps including the following IPV item sets: (1) four item sets (physical IPV, sexual IPV, psychological IPV, controlling behaviors) to assess the invariance of a four-dimensional IPV model, (2) three item sets (physical IPV, sexual IPV, controlling behaviours) to assess the invariance of a three-dimensional IPV model, and (3) two item sets (physical IPV and either sexual IPV or controlling behaviors) to assess the invariance of a bidimensional IPV model. We encountered challenges completing all analytical steps for these models (Supplemental File S1), which we discuss in the Limitations section of the Discussion with recommendations for future research. To address these challenges, we applied the above analytical steps to assess the invariance of unidimensional IPV models for item sets that arguably were more behaviourally based and/or more content validity because they included more items [34]. So, the analyses presented in the body of this paper assessed separately the measurement invariance of the seven physical-IPV items and the five controlling-behaviour items.

## Results

### Conventional prevalence estimates of IPV

Estimates for lifetime IPV were generally high but ranged widely across sample countries (Table 2). Reported lifetime experience of physical IPV ranged from 5.6% in Comoros to 50.5% in Afghanistan. Reported lifetime experience of sexual IPV ranged from 1.1% in Armenia to 25.5% in the DRC. Reported lifetime experience of psychological IPV ranged from 6.4% in Comoros to 50.8% in Afghanistan, and reported experiences of controlling behaviours ranged from 25.9% in Cambodia to 84.7% in Gabon.

**Table 2:** National (weighted) estimates for lifetime and prior-year intimate partner violence, 36 Demographic and Health Surveys across 36 countries (2012–2018)

	Lifetime					Prior-Year					Controlling Behaviour (any)
Country	Psych.	Phys.	Sexual	Phys. / Sexual	Any	Psych.	Phys.	Sexual	Phys. / Sexual	Any	
**Central Asia**											
Kyrgyz Republic	14.1	25.1	4.0	25.4	28.1	10.4	16.9	2.8	17.1	19.8	81.9
Tajikistan	15.8	25.3	1.7	25.7	30.8	13.3	18.7	1.4	19.0	24.1	80.7
**Latin America, Caribbean**											
Haiti	26.3	18.6	11.2	23.5	34.0	17.8	10.0	7.0	13.8	22.3	72.6
**N Africa, W Asia, Europe**											
Armenia	11.4	8.0	1.1	8.1	14.0	6.4	3.5	0.3	3.5	7.6	49.4
Egypt	18.8	25.2	4.1	25.6	30.3	13.1	13.5	2.7	14.0	18.6	78.0
**S, SE Asia**											
Afghanistan	37.3	50.5	7.5	50.8	55.5	34.4	45.8	6.1	46.0	51.8	68.8
Cambodia	24.8	16.2	5.5	18.2	28.7	17.3	9.3	3.9	10.9	19.6	25.9
India	13.8	29.8	7.0	30.9	33.3	11.4	22.5	5.7	23.9	26.5	46.1
Maldives	11.6	12.4	2.0	12.6	17.8	7.6	5.4	0.7	5.5	10.4	38.3
Myanmar	13.5	15.4	3.0	16.3	20.9	10.2	10.2	2.2	11.0	15.0	29.1
Nepal	12.3	22.8	7.0	24.3	26.3	7.7	10.0	4.0	11.2	13.5	34.3
Pakistan	25.8	22.9	4.8	23.7	33.5	20.6	13.6	3.6	14.5	24.8	28.1
Philippines	10.7	11.0	4.0	12.2	16.5	6.6	4.3	2.2	5.4	9.0	37.5
Timor-Leste	9.4	36.6	5.0	38.1	40.1	8.9	33.1	4.8	34.6	36.8	47.3
**Sub-Saharan Africa**											
Angola	27.7	32.5	7.7	33.9	41.3	24.0	24.2	6.7	25.8	33.8	55.9
Benin	36.7	19.5	8.8	22.4	41.8	28.7	11.1	6.1	13.9	31.8	65.3
Burundi	25.6	39.7	25.4	46.7	50.2	16.5	17.9	18.4	27.8	31.6	35.4
Chad	24.1	26.4	10.0	28.6	34.8	16.3	15.5	6.8	17.4	23.1	66.2
Comoros	8.1	5.6	1.8	6.4	10.6	6.2	4.2	1.3	4.8	8.1	66.8
DRC	36.6	45.9	25.5	50.7	57.4	29.4	30.3	19.8	36.7	43.9	82.7
Ethiopia	24.0	23.5	10.1	26.3	33.8	20.2	16.9	8.3	19.8	27.0	56.7
Gabon	35.1	46.2	17.0	48.6	56.1	26.6	28.3	11.8	31.2	39.2	84.7
Gambia	15.8	19.6	2.7	20.1	26.2	8.5	6.9	1.1	7.3	12.3	51.2
Kenya	32.4	36.9	13.3	39.4	47.1	23.8	22.6	9.8	25.4	32.7	63.2
Malawi	29.5	25.9	19.2	33.8	42.2	23.0	16.2	15.4	24.1	32.6	71.4
Mali	38.4	36.8	11.8	38.5	48.9	28.1	18.0	7.8	20.9	34.0	63.7
Mozambique	14.9	18.1	3.6	18.8	23.5	12.2	14.1	2.9	14.7	18.8	39.7
Namibia	25.0	23.4	7.6	25.0	33.3	21.0	18.7	6.6	20.2	27.8	52.3
Nigeria	19.2	14.4	4.8	16.2	24.5	15.3	9.3	3.7	11.0	19.0	63.9
Rwanda	26.6	31.1	11.6	34.4	40.4	18.5	17.6	8.3	20.6	26.7	44.9
Sierra Leone	29.2	44.2	7.3	45.3	50.5	20.8	27.2	5.1	28.6	33.9	79.2
Tanzania	35.9	39.3	13.6	41.7	49.5	28.1	27.0	10.4	29.5	37.5	74.2
Togo	29.7	20.2	7.5	22.1	35.7	24.1	10.7	4.8	12.7	27.2	64.5
Uganda	41.1	40.1	22.9	46.6	55.8	29.3	22.3	16.4	29.6	39.4	71.4
Zambia	24.0	38.8	16.7	42.7	47.1	17.8	21.3	13.0	26.5	31.1	73.8
Zimbabwe	31.5	30.7	12.7	35.4	45.0	23.5	15.2	9.3	19.8	30.1	66.4
Max	41.1	50.5	25.5	50.8	57.4	34.4	45.8	19.8	46.0	51.8	84.7
Min	8.1	5.6	1.1	6.4	10.6	6.2	3.5	0.3	3.5	7.6	25.9

*Abbreviations*: *DRC* Democratic Republic of Congo, *Psych.* psychological, *Phys.* physical

Reported prior-year prevalences of IPV, by type, also are presented in Table 2. In general, the lower item-specific prevalences for prior-year IPV, by type, made invariance testing with these measures more difficult (Supplemental File S1; results available on request).

### Results from country-specific exploratory and confirmatory factor analyses

Tables 3 and 4 present the results for country-specific EFAs and CFAs for lifetime physical IPV (Table 3) and for controlling behaviours (Table 4) for all 36 DHS samples. For lifetime physical IPV, across all countries, all loadings exceeded 0.55 in the country-specific EFAs and exceeded 0.65 in the country-specific CFAs, above the 0.35 recommended benchmark. Moreover, all model-fit statistics (RMSEA, CFI, TLI) were within recommended benchmarks (Table 3). For controlling behaviours, across all countries, all loadings exceeded 0.50 in the country-specific EFAs and exceeded 0.40 in the country-specific CFAs, above the 0.35 recommended benchmark. Moreover, in almost all cases, model-fit statistics (RMSEA, CFI, TLI) were within recommended benchmarks (Table 4). Thus, in country-specific EFAs and CFAs, unidimensional models for the seven physical-IPV items and the five controlling-behaviour items had reasonable fits with the data across all countries. The country-specific loadings for each item and the ranges of estimated item loadings across countries are reported in Supplemental Tables S3a and S3b.

**Table 3:** Results of country-specific factor analyses and alignment optimization cross-country measurement invariance analysis, seven lifetime physical intimate partner violence items, *N* = 36 Demographic and Health Surveys across 36 countries (2012–2018)

	Country-Specific EFAs^a^ (*N* = 36)				Country-Specific CFAs^a^ (*N* = 36)				Alignment Optimization^b^
Country	Loadings	RMSEA	CFI	TLI	Loadings	RMSEA	CFI	TLI	Non-invariant parameters (intercepts, loadings)
**Central Asia**									
Kyrgyz Republic	0.84–0.95	0.02	1.00	1.00	0.90–0.96	0.02	1.00	1.00	2,0
Tajikistan	0.65–0.95	0.02	1.00	1.00	0.74–1.00	0.06	0.99	0.99	0,0
**Latin America and the Caribbean**									
Haiti	0.70–0.97	0.00	1.00	1.00	0.67–0.94	0.02	1.00	1.00	3,0
**North Africa, West Asia, Europe**									
Armenia	0.95–0.98	0.00	1.00	1.00	0.95–1.00	0.00	1.00	1.00	3,1
Egypt	0.83–0.97	0.02	1.00	1.00	0.87–0.96	0.03	1.00	1.00	2,0
**South and Southeast Asia**									
Afghanistan	0.89–0.96	0.03	0.99	0.98	0.86–0.97	0.03	0.99	0.98	1,0
Cambodia	0.60–0.94	0.02	1.00	1.00	0.82–0.96	0.00	1.00	1.00	2,1
India	0.78–0.94	0.02	1.00	0.99	0.81–0.93	0.03	0.99	0.99	1,0
Maldives	0.94–0.98	0.01	1.00	1.00	0.73–0.99	0.00	1.00	1.00	1,0
Myanmar	0.78–0.97	0.01	1.00	1.00	0.78–0.97	0.02	1.00	1.00	2,0
Nepal	0.84–0.97	0.02	1.00	1.00	0.84–0.98	0.01	1.00	1.00	1,0
Pakistan	0.86–0.97	0.02	1.00	1.00	0.85–0.98	0.05	0.99	0.99	0,1
Philippines	0.89–0.97	0.01	1.00	1.00	0.82–0.95	0.01	1.00	1.00	4,1
Timor-Leste	0.66–0.92	0.03	0.99	0.98	0.66–0.93	0.03	0.99	0.99	1,0
**Sub-Saharan Africa**									
Angola	0.81–0.93	0.03	1.00	0.99	0.78–0.94	0.02	1.00	0.99	1,0
Benin	0.81–0.97	0.03	1.00	1.00	0.88–0.93	0.02	1.00	0.99	1,0
Burundi	0.84–0.93	0.02	1.00	1.00	0.76–0.93	0.03	1.00	0.99	2,0
Chad	0.85–0.95	0.04	0.99	0.99	0.82–0.94	0.01	1.00	1.00	0,0
Comoros	0.74–0.99	0.02	1.00	0.99	0.82–1.00	0.01	1.00	1.00	1,0
DRC	0.80–0.87	0.02	0.99	0.99	0.75–0.91	0.03	0.99	0.98	0,0
Ethiopia	0.76–0.92	0.02	1.00	1.00	0.80–0.95	0.02	1.00	0.99	0,0
Gabon	0.64–0.95	0.03	1.00	1.00	0.85–0.98	0.06	0.99	0.99	3,1
Gambia	0.57–0.96	0.01	1.00	1.00	0.83–1.00	0.02	0.99	0.98	2,0
Kenya	0.79–0.94	0.03	1.00	1.00	0.82–0.93	0.02	1.00	1.00	1,0
Malawi	0.83–0.94	0.03	1.00	0.99	0.83–0.94	0.00	1.00	1.00	3,0
Mali	0.58–0.91	0.01	1.00	1.00	0.74–0.86	0.00	1.00	1.00	1,0
Mozambique	0.83–0.95	0.03	1.00	1.00	0.74–0.96	0.01	1.00	1.00	2,0
Namibia	0.87–0.98	0.03	1.00	1.00	0.86–0.98	0.02	1.00	1.00	1,0
Nigeria	0.78–0.96	0.01	1.00	1.00	0.74–0.96	0.02	1.00	1.00	2,1
Rwanda	0.83–0.95	0.02	1.00	1.00	0.84–0.95	0.02	1.00	1.00	1,0
Sierra Leone	0.74–0.95	0.03	0.99	0.99	0.74–0.95	0.04	0.99	0.98	1,0
Tanzania	0.76–0.93	0.02	1.00	1.00	0.80–0.93	0.01	1.00	1.00	2,1
Togo	0.82–0.95	0.02	1.00	1.00	0.85–0.95	0.03	1.00	0.99	1,0
Uganda	0.74–0.93	0.02	1.00	1.00	0.75–0.94	0.02	1.00	1.00	3,0
Zambia	0.86–0.91	0.02	1.00	1.00	0.79–0.93	0.02	1.00	1.00	1,0
Zimbabwe	0.77–0.93	0.02	1.00	1.00	0.79–0.94	0.02	1.00	1.00	3,1

*DRC* Democratic Republic of Congo

^a^Model fit criteria exploratory, confirmatory factor analysis (EFA, CFA): root mean square error of approximation (RMSEA) ≤0·08, comparative fit index (CFI) ≥0·95, Tucker-Lewis index (TLI) ≥0.95, loadings ≥0.35

^b^Alignment optimization model fit criteria: < 25% of model estimates non-invariant. Each country has 14 parameter estimates (7 intercepts, 7 loadings)

**Table 4:** Results of country-specific factor analyses and alignment optimization cross-country measurement invariance analysis, five controlling behaviour items, *N* = 36 Demographic and Health Surveys across 36 countries (2012–2018)

	Country-Specific EFAs^a^ (*N* = 36)				Country-Specific CFAs^a^ (*N* = 36)				Alignment Optimization^b^
Country	Loadings	RMSEA	CFI	TLI	Loadings	RMSEA	CFI	TLI	Non-invariant parameters (intercepts, loadings)
**Central Asia**									
Kyrgyz Republic	0.62–0.97	0.08	0.97	0.93	0.63–0.99	0.07	0.95	0.91	2,0
Tajikistan	0.68–0.84	0.07	0.95	0.91	0.65–0.90	0.06	0.95	0.91	1,0
**Latin America and the Caribbean**									
Haiti	0.74–0.91	0.06	0.99	0.98	0.76–0.89	0.08	0.97	0.94	1,0
**North Africa, West Asia, Europe**									
Armenia	0.77–0.92	0.04	0.99	0.98	0.77–0.86	0.02	0.99	0.99	0,0
Egypt	0.55–0.79	0.07	0.92	0.83	0.41–0.74	0.04	0.94	0.87	2,0
**South and Southeast Asia**									
Afghanistan	0.66–0.85	0.02	0.98	0.95	0.71–0.84	0.02	0.98	0.96	0,0
Cambodia	0.77–0.95	0.04	1.00	0.99	0.82–0.94	0.04	1.00	0.99	0,0
India	0.72–0.85	0.03	0.95	0.91	0.74–0.86	0.03	0.95	0.89	3,1
Maldives	0.77–0.92	0.02	1.00	0.99	0.67–0.95	0.03	0.99	0.99	0,0
Myanmar	0.73–0.91	0.03	0.99	0.98	0.69–0.91	0.07	0.97	0.93	0,0
Nepal	0.75–0.89	0.06	0.99	0.98	0.72–0.89	0.04	0.99	0.99	1,1
Pakistan	0.79–0.90	0.02	1.00	1.00	0.80–0.95	0.07	0.97	0.94	1,0
Philippines	0.77–0.90	0.03	0.99	0.98	0.79–0.89	0.02	1.00	0.99	0,0
Timor-Leste	0.72–0.93	0.05	0.98	0.96	0.66–0.90	0.02	0.99	0.99	1,0
**Sub-Saharan Africa**									
Angola	0.81–0.86	0.04	0.99	0.99	0.76–0.87	0.05	0.98	0.97	0,0
Benin	0.74–0.84	0.06	0.97	0.93	0.76–0.83	0.06	0.97	0.93	0,0
Burundi	0.81–0.91	0.07	0.99	0.97	0.87–0.90	0.07	0.99	0.98	3,0
Chad	0.70–0.88	0.05	0.99	0.98	0.76–0.90	0.06	0.99	0.97	0,0
Comoros	0.77–0.87	0.10	0.97	0.93	0.75–0.90	0.08	0.99	0.98	0,0
DRC	0.70–0.80	0.04	0.98	0.95	0.71–0.79	0.05	0.97	0.94	0,0
Ethiopia	0.53–0.87	0.03	0.98	0.97	0.50–0.85	0.03	0.99	0.98	1,1
Gabon	0.60–0.86	0.05	0.98	0.96	0.66–0.88	0.08	0.98	0.96	0,0
Gambia	0.63–0.95	0.07	0.93	0.86	0.75–0.92	0.07	0.97	0.94	0,0
Kenya	0.76–0.86	0.05	0.99	0.98	0.78–0.85	0.03	1.00	0.99	0,0
Malawi	0.71–0.84	0.08	0.96	0.92	0.70–0.84	0.06	0.98	0.96	2,0
Mali	0.77–0.85	0.08	0.98	0.95	0.74–0.88	0.06	0.98	0.97	0,0
Mozambique	0.83–0.91	0.03	1.00	0.99	0.82–0.92	0.04	1.00	0.99	0,0
Namibia	0.82–0.93	0.04	1.00	0.99	0.82–0.95	0.03	1.00	1.00	0,0
Nigeria	0.63–0.87	0.03	0.98	0.96	0.67–0.89	0.03	0.98	0.96	1,0
Rwanda	0.84–0.92	0.08	0.99	0.98	0.78–0.87	0.05	0.99	0.99	0,0
Sierra Leone	0.63–0.91	0.08	0.96	0.92	0.70–0.85	0.09	0.96	0.92	0,0
Tanzania	0.72–0.83	0.04	0.99	0.97	0.74–0.85	0.06	0.97	0.95	0,0
Togo	0.76–0.85	0.06	0.98	0.96	0.71–0.85	0.03	0.99	0.97	1,0
Uganda	0.78–0.87	0.07	0.97	0.94	0.76–0.82	0.08	0.95	0.91	0,0
Zambia	0.72–0.87	0.06	0.98	0.95	0.73–0.89	0.06	0.97	0.94	0,0
Zimbabwe	0.76–0.89	0.07	0.98	0.96	0.74–0.93	0.10	0.96	0.92	1,0

*DRC* Democratic Republic of Congo

^a^Model fit criteria exploratory, confirmatory factor analysis (EFA, CFA): root mean square error of approximation (RMSEA) ≤0·08, comparative fit index (CFI) ≥0·95, Tucker-Lewis index (TLI) ≥0.95, loadings ≥0.35

^b^Alignment optimization model fit criteria: < 25% of model estimates non-invariant. Each country has 10 parameter estimates (5 intercepts, 5 loadings)

### Multiple-group CFA results: assessment of exact measurement invariance

Table 5 presents results for the MGCFAs for physical IPV (Panel 1) and controlling behaviours (Panel 2), across all 36 included countries. For the physical-IPV unidimensional model, the metric and configural models differed significantly (at *p* < 0.001), as did the scalar and metric models (at *p* < 0.001). Based on the test statistics and their proposed benchmarks, metric invariance across countries was not achieved. Similarly, for the controlling-behaviour unidimensional model, the metric and configural models differed significantly (at *p* < 0.001), as did the scalar and metric models (at *p* < 0.001). Based on the test statistics and their proposed benchmarks, metric invariance across countries was not achieved.

**Table 5:** Multiple-group confirmatory factor analysis, *N* = 136,693 across Demographic and Health Surveys in 36 countries, 2012–2018

Model	Loglikelihood	Number of parameters	Models compared	Chi-square	Degrees of freedom	*P* value
Panel 1: Seven physical-IPV items						
Configural	− 462,468.524	539				
Metric	− 463,664.754	364	Metric against Configural	1089.52418	175	<.001
Scalar	− 466,670.413	119	Scalar against Metric	4511.56926	245	<.001
Panel 2: Five controlling-behavior items						
Configural	−547332	395				
Metric	−547971	290	Metric against Configural	1277.248	105	<.001
Scalar	−559118	115	Scalar against Metric	22294.300	175	<.001

### Alignment optimization results: assessment of approximate measurement invariance

Given the lack of *exact measurement invariance* based on the MGCFA results, Table 6 presents the results based on alignment optimization, in which we assessed approximate measurement invariance separately for the physical-IPV items (Panel 1) and the controlling-behaviour items (Panel 2). For physical IPV, 55 (or 21.8% of) estimated thresholds, eight (or 2.8% of) estimated loadings, and 12.3% of all parameter estimates were measurement non-invariant (Table 3). The items ‘slap’, ‘choke’, and ‘twist’ had a low degree of threshold invariance, and the item ‘choke’ had a low degree of loading invariance (see low R^2^ values Table 6, Panel 1). For controlling behaviours, 21 (or 11.7% of) estimated thresholds, three (or 1.7% of) estimated loadings, and 6.7% of all parameter estimates were measurement non-invariant (Table 4). All items had a reasonable degree of threshold invariance; however, the items ‘meet your female friends’ and ‘contact with your family’ had a low degree of loading invariance (see low R^2^ values in Table 6, Panel 2). Again, a guideline of 25% or fewer total non-invariant parameter estimates is recommended for trustworthy latent mean estimates and their comparison across groups. Overall, results suggested that the DHS item sets for physical IPV and controlling behaviours exhibited approximate measurement invariance across the 36 countries and allowed acceptable alignment performance.

**Table 6:** Results from alignment optimization analysis, *N* = 136,693 across Demographic and Health Surveys in 36 countries, 2012–2018

	Thresholds		Loadings	
Items	Weighted Average Value across Invariant Groups	R^2^	Weighted Average Value across Invariant Groups	R^2^
Panel 1: Seven physical-IPV items				
Push you, shake you, or throw something at you?	2.081	0.351	2.915	0.836
Slap you?	0.263	0.000	3.867	0.394
Punch with his fist or with something that could hurt you?	3.676	0.715	3.614	0.683
Kick you, drag you, or beat you up?	3.645	0.381	3.474	0.213
Try to choke you or burn you on purpose?	5.882	0.073	2.806	0.051
Threaten to attack you with a knife, gun or other weapon?	6.056	0.634	2.248	0.469
Twist your arm or pull your hair?	3.220	0.000	3.599	0.359
Panel 2: Five controlling-behaviour items				
Jealous or angry if you talk/talked to other men?	-0.621	0.753	1.754	0.387
Frequently accuses/accused you of being unfaithful?	1.690	0.704	2.424	0.443
Does/did not permit you to meet your female friends?	1.924	0.375	2.759	0.000
Tries/tried to limit your contact with your family?	3.016	0.527	2.601	0.000
Insists/insisted on knowing where you are/were at all times?	0.100	0.629	2.061	0.529

### Country rankings on level of physical IPV based on AO-estimates and standard prevalence

**Fig. 1:**
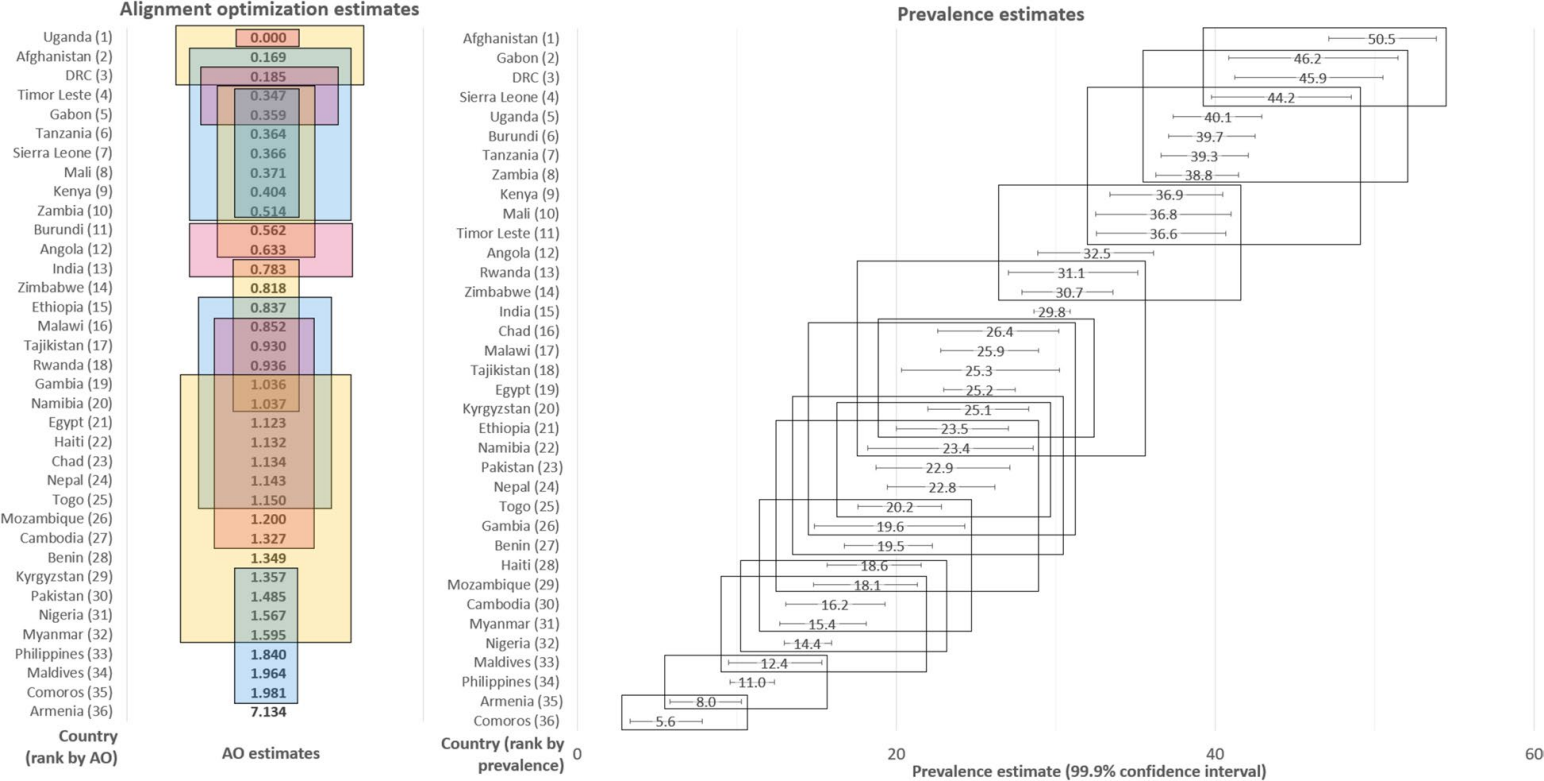
Levels of physical IPV derived from the alignment optimization approach and conventional prevalence estimation and associated country rankings, *N* = 36 Demographic and Health Surveys for 36 countries from 2012 to 2018

For illustration, Fig. 1 compares country rankings on level of lifetime physical IPV based on AO-derived scores versus conventional prevalence estimates. (Full country-ranking results for physical-IPV and analogous results for controlling behaviours are available on request.) The physical IPV scores are factor means derived from the final AO factor model, which presumes that observed items reflect a latent physical IPV construct. The prevalence estimates are based on aggregates of the observed responses to physical IPV items using mean estimation with adjustment for sampling. Uncertainties in both sets of estimates are reflected in 99.9% confidence intervals to account for multiple comparisons. As shown in Fig. 1, the distributions of country rankings based on AO-derived scores and prevalence estimates suggested some country-level differences; however, a Wilcoxon matched-pairs sign-rank test supported no significant difference in country rankings. Both sets of estimates exhibited a high degree of clustering. For example, in comparing countries using AO-derived scores, 12 clusters emerged, wherein country estimates did not differ significantly from one another. In comparing countries by conventional estimates of prevalence and associated confidence limits, three major clusters emerged: countries ranked 1–12, those ranked 13–30, and those ranked 31–36.

### Convergent validity of AO-derived scores for physical IPV and controlling behaviour with IPV prevalences

As expected, AO-derived scores for physical IPV and for controlling behaviours were positively correlated with prevalence estimates for all four types of IPV, providing evidence for convergent validity. Pairwise correlations for physical IPV ranged from 0.48 to 0.66, and those for controlling behaviour ranged from 0.49 to 0.87. Pairwise scatter plots provided empirical support for linear relationships (Supplemental Fig. S1).

## Discussion

### Summary of findings

This is the first cross-national analysis to assess the measurement invariance of seven standard physical-IPV items and five standard controlling-behaviour items from the DVM administered in 36 DHS across 36 LMICs during 2012–2018. Included countries spanned five world regions and had populations that varied in size, schooling attainment for women, income inequality, and degree of gender equity in the national legal environment. Elements of survey administration related to team size, number of training days, and average interview duration also differed across countries.

In separate (unidimensional) analyses, both item sets exhibited good country-specific measurement properties for all 36 LMICs. Although neither item set met the criteria for metric or scalar invariance, both item sets did meet the criteria for approximate invariance across all 36 LMICs. The distributions of country rankings, based on AO-derived scores and conventional prevalence estimates, were similar for physical IPV and for controlling behaviours. However, both AO-derived scores and prevalence estimates often were highly clustered and not significantly different, suggesting that individual country rankings were not interpretable using either set of estimates for either type of IPV.

### Limitations and strengths

Findings are limited to the seven physical-IPV items and five controlling-behaviour items included in this analysis. As such, findings cannot be extrapolated to different physical IPV items, different controlling-behaviour items, or other item sets intended to capture other types of IPV. This limitation is important, given the challenges we encountered when attempting to undertake the same analytical steps for other combinations of IPV items sets (Supplemental File S1). These analytical challenges may be attributable to a variety of issues. First, the conceptualizations of psychological IPV [34–36] and sexual IPV [34] remain under-developed, especially in research undertaken in LMIC settings. Second, the sexual IPV and psychological IPV items sets used in the DHS each included only three items capturing a narrow subset of behaviors. The sexual IPV items, for example, captured only “forced” sex acts and excluded acts that occur when the victim is unable to consent [34]. Small item sets that lack content validity may miss acts that contribute importantly to the latent construct across countries. Third, low item-specific prevalences (especially for sexual IPV) have been noted as a concern for efforts to validate measures of IPV [37]. In our case, low item prevalences prevented model convergence during the Monte Carlo simulation stage of some AO analyses. Underestimates of IPV present ongoing challenges to the accurate measurement of IPV in LMICs [38]. For the DHS DVM, some of this low prevalence may have arisen because the DVM is implemented at the end of a sometimes long, multi-purpose survey (Table 1), when respondents and interviewers may be fatigued. Fourth, the less behaviourally-based and more subjective nature of the sexual IPV items (e.g., “physically forced”) and psychological IPV items (“humiliated”) may be a source of non-invariance, as such wording may be interpreted differently across languages and contexts. Finally, some differences in survey administration across countries in this analysis (e.g., team size; training duration; interview duration) may have contributed to our inability to establish exact invariance for items in the analysis. The DVM typically is administered at or toward the end of the women’s interview; therefore, the inclusion of more, sensitive, or different modules earlier in the interview may have framed the DVM in ways that affected its measurement invariance across countries.

Findings also are limited to this non-representative set of LMICs and for the period of analysis (2012–2018). Nevertheless, the establishment of approximate measurement invariance for seven physical-IPV items and five controlling-behaviour items across highly diverse LMICs spanning five world regions suggests the utility of these item sets to compare countries on these dimensions of IPV. These results support their use to monitor SDG5.2.1.

### Implications for research and policy

Findings from this analysis have two major implications for future research and policy. First, we recommend that this analysis be replicated for high-income countries (HICs), LMICs in regions that are under-represented here, and surveys conducted before or after 2012–2018. Second, many of the estimates for physical IPV and controlling behaviours–whether derived from alignment optimization or based on standard prevalences–were not statistically different, when using a more conservative *p*-value (< 0.001) that took multiple comparisons into account. If national-level comparisons of estimates for physical IPV and controlling behaviours are a priority for monitoring SDG5.2.1, we recommend that such comparisons be based on estimates derived from larger national samples, which helps to reduce sampling error and to increase statistical power. By extension, any cross-time comparison of national IPV estimates between independent, repeated cross-sectional surveys may require larger sample sizes. Finally, if resource constraints do not allow sample surveys to be designed to reduce sampling error, we recommend that an international body like the World Health Organization consider convening an expert panel to deliberate the utility and policy relevance of establishing ranges for physical IPV and controlling behaviour that permit grouped comparisons.

Third, the physical IPV items ‘slap,’ ‘twist’, and ‘choke’ exhibited a low degree of intercept and/or loading invariance across countries. Cognitive testing of these items is recommended to improve their cross-national measurement equivalence. Likewise, the controlling-behaviour items ‘…meet your female friends’ and ‘…contact with your family’ exhibited a low degree of loading invariance across countries, and cognitive testing of these items also is recommended to improve their cross-national psychometric performance.

Fourth, further testing of these item sets for measurement invariance across repeated national surveys is needed to assess how invariant these item sets are over extended periods of time. Fifth, this analysis should be replicated for expanded psychological-IPV and sexual-IPV item sets, both currently only three items each. Until then, the seven physical-IPV items and the five controlling-behaviour items from the DHS DVM appear useful to measure and to compare countries on levels of IPV against women.

## Conclusion

Alignment Optimization is a powerful approach to assess approximate measurement equivalence of IPV scales across widely diverse countries charged with monitoring SDG5.2. The seven physical-IPV items and the five controlling-behaviour items from the DHS DVM exhibit approximate measurement invariance across 36 diverse LMICs spanning five regions. If shown to be invariant over calendar time and across HICs, these item sets may be useful to monitor SDG5.2 globally.

## Supplementary Information

**Additional file 1.** Monitoring SDG 5 Supplementary Materials.docx contains supplemental tables and figures referenced in the text.

## Data Availability

Data from the Demographic and Health Surveys (DHS) are publicly available upon reasonable request to Measure DHS: https://dhsprogram.com/data/new-user-registration.cfm. Investigators must request from Measure DHS access to the data used here.

## Abbreviations

IPV: Intimate Partner Violence
CI: Confidence Interval
GDP: Gross Domestic Product
SDG: Sustainable Development Goal
WHO: World Health Organization
DHS: Demographic and Health Surveys
DVM: Domestic Violence Module
LMIC: Lower- and Middle-Income Country
VAWG: Violence Against Women and Girls
USD: United States Dollar
EFA: Exploratory Factor Analysis
RMSEA: Root Mean Square Error of Approximation
CFI: Comparative Fit Index
TLI: Tucker-Lewis Index
CFA: Confirmatory Factor Analysis
MGCFA: Multiple-Group Confirmatory Factor Analysis
AO: Alignment Optimization
DRC: Democratic Republic of the Congo
HIC: High-Income Country

## References

[1] World Health Organization on behalf of the United Nations Inter-Agency Working Group on Violence Against Women Estimation and Data (UNICEF, U., UNODC, UNSD, UNWomen), Violence against women prevalence estimates 2018 (2021). Global, regional and national prevalence estimates for intimate partner violence against women and global and regional prevalence estimates for non-partner sexual violence against women.

[2] Devries KM (2013). Intimate partner violence and incident depressive symptoms and suicide attempts: a systematic review of longitudinal studies. PLoS Med.

[3] Devries KM (2014). Intimate partner violence victimization and alcohol consumption in women: a systematic review and meta-analysis. Addiction.

[4] Dillon G (2013). Mental and physical health and intimate partner violence against women: a review of the literature. Int J Family Med.

[5] Crane CA, Hawes SW, Weinberger AH (2013). Intimate partner violence victimization and cigarette smoking: a meta-analytic review. Trauma Violence Abuse.

[6] Beydoun HA (2012). Intimate partner violence against adult women and its association with major depressive disorder, depressive symptoms and postpartum depression: a systematic review and meta-analysis. Soc Sci Med.

[7] Maxwell L (2015). Estimating the effect of intimate partner violence on women's use of contraception: a systematic review and meta-analysis. PLoS One.

[8] Yount KM (2005). Resources, family organization, and domestic violence against married women in Minya. Egypt J Marriage Fam.

[9] Potter LC (2020). Categories and health impacts of intimate partner violence in the World Health Organization multi-country study on women’s health and domestic violence. Int J Epidemiol.

[10] Hoeffler A, Fearon J (2014). Benefits and costs of the conflict and violence targets for the post-2015 development agenda, in Post-2015 consensus, conflict and violence assessment paper.

[11] United Nations Department of Economic and Social Affairs Statistics Division (2014). Guidelines for producing statistics on violence against women-statistical surveys.

[12] United Nations (2015). Transforming our world: the 2030 agenda for sustainable development.

[13] Devries KM (2013). Global health. The global prevalence of intimate partner violence against women. Science.

[14] Garcia-Moreno C (2006). Prevalence of intimate partner violence: findings from the WHO multi-country study on women's health and domestic violence. Lancet.

[15] Kishor S, Johnson K (2004). Profiling domestic violence: a multi-country study.

[16] Breiding MJ (2015). Intimate partner violence surveillance: uniform definitions and recommended data elements, version 2.0.

[17] World Health Organization (2005). WHO multi-country study on women’s health and domestic violence against women: summary report of initial results onprevalence, health outcomes and women’s responses.

[18] MEASURE DHS and ICF International (2014). Domestic violence module: demographic and health surveys methodology.

[19] Short Fabic M, Choi Y, Bird S (2012). A systematic review of demographic and health surveys: data availability and utilization for research. Bull World Health Organ.

[20] Hancioglu A, Arnold F (2013). Measuring coverage in MNCH: tracking progress in health for women and children using DHS and MICS household surveys. PLoS Med.

[21] Yount KM (2011). Response effects to attitudinal questions about domestic violence against women: a comparative perspective. Soc Sci Res.

[22] Guenole N, Brown A (2014). The consequences of ignoring measurement invariance for path coefficients in structural equation models. Front Psychol.

[23] Costa D, Barros H (2016). Instruments to assess intimate partner violence: a scoping review of the literature. Violence Vict.

[24] Ellsberg M, Heise L (2002). Bearing witness: ethics in domestic violence research. Lancet.

[25] StataCorp (2019). Stata statisical software: release 16.

[26] Muthén LK, Muthén BO. Mplus user's guide. 8th ed. null. 1998-2017. Los Angeles: Muthén & Muthén.

[27] Hu L, Bentler PM (1999). Cutoff criteria for fit indexes in covariance structure analysis: conventional criteria versus new alternatives. Struct Equ Model Multidiscip J.

[28] Brown TA (2006). Confirmatory factor analysis for applied research.

[29] Vandenberg RJ, Lance CE. A review and synthesis of the measurement invariance literature: suggestions, practices, and recommendations for organizational research. Organ Res. 2000;3(1):4–70.

[30] Muthén B, Asparouhov T (2014). IRT studies of many groups: the alignment method. Front Psychol.

[31] Davidov E, et al. The comparability of measurements of attitudes toward immigration in the European social survey: exact versus approximate measurement equivalence. Public Opin Q. 2015;79(S1):244–66.

[32] Muthén B, Asparouhov T (2018). Recent methods for the study of measurement invariance with many groups: alignment and random effects. Sociol Methods Res.

[33] Asparouhov T, Muthén B (2014). Multiple-group factor analysis alignment. Struct Equ Model Multidiscip J.

[34] Follingstad DR, Rogers MJJSR. Validity concerns in the measurement of women’s and men’s report of intimate partner violence. Sex Roles. 2013;69(3–4):149–67.

[35] Martín-Fernández M, Gracia E, Lila MJBPH. Psychological intimate partner violence against women in the European Union: a cross-national invariance study. BMC Public Health. 2019;19(1):1–11. 10.1186/s12889-019-7998-0 PMC6935167 31881950

[36] Porrúa-García C, et al. Development and validation of the scale of psychological abuse in intimate partner violence (EAPA-P). Psicothema. 2016;28(2):214–21. 10.7334/psicothema2015.197 27112821

[37] Ryan KMJSr. Issues of reliability in measuring intimate partner violence during courtship. Sex Roles. 2013;69(3–4):131–48.

[38] Palermo T, Bleck J, Peterman AJAjoe. Tip of the iceberg: reporting and gender-based violence in developing countries. Am J Epidemiol. 2014;179(5):602–12. 10.1093/aje/kwt295 PMC3927971 24335278

