# Supplementary material for: Global measurement of intimate partner violence to monitor Sustainable Development Goal 5
Source: BMC Public Health. 2022 Mar 8;22:465. doi: 10.1186/s12889-022-12822-9 (PMC8903149; doi:10.1186/s12889-022-12822-9)
Supplement: Supplementary file 1 — Additional file 1. Monitoring SDG 5 Supplementary Materials.docx contains supplemental tables and figures referenced in the text. [file 12889_2022_12822_MOESM1_ESM.pdf]

Supplementary Materials for

Global Measurement of Intimate Partner Violence to Monitor Sustainable Development Goal 5

Authors: Kathryn M. Yount, PhD, Yuk Fai Cheong, PhD, Zara Khan, MPH, Irina Bergenfeld, MPH, Nadine Kaslow, PhD, Cari Jo Clark, PhD

Correspondence to: kyount@emory.edu

This file includes:

File S1. Summary of all completed analyses
Table S1. Items included in the World Bank's Women, Business and the Law Index (Women, Business, and Law Data for 2016)
Table S2. Items included in the DHS Domestic Violence module
Table S3a. Item loadings from exploratory and confirmatory factor analyses of physical intimate partner violence, N=36 Demographic and Health Surveys across 36 countries (2012-2018)
Table S3b. Item loadings from exploratory and confirmatory factor analyses of controlling behaviour, N=36 Demographic and Health Surveys across 36 countries (2012-2018)
Figure S1. Correlations of prevalence estimates and alignment optimization estimates of lifetime intimate partner violence across 35 countries (outlier removed), 2012-2018
Figure S2. Correlations of prevalence estimates and alignment optimization estimates of controlling behaviour across 36 countries, 2012-2018.

File S1. Summary of all completed analyses.

The results of all supplemental analyses are available upon request to the corresponding author.

Data Overview. The Demographic and Health Surveys (DHS) Domestic Violence Module contains 18 items measuring lifetime experiences of intimate partner violence (IPV), of which seven items assess experiences of physical IPV, three items assess experiences of sexual IPV, three items assess experiences of psychological IPV, and five items assess experiences of controlling behaviors.

Step 1. Descriptive analyses were completed for all items across all countries (N=36) that had all 18 items and a national DHS conducted between 2012 and 2018, with results reported in Main Table 2.

Step 2. Following descriptive analyses, for each country, exploratory factor analysis (EFA) was performed to explore dimensionality of the IPV construct, magnitudes of the factor loadings, and overall fit of models incorporating all 18 items. EFA models were considered to have adequate fit to the data if all item factor loadings had a magnitude of 0.35 or greater, and overall measures of model fit met the following general guidelines: the root mean square error of approximation (RMSEA) was ≤0.08, the comparative fit index (CFI) was ≥0.95, and the Tucker-Lewis index (TLI) was ≥0.95 [1]. Results indicated inadequate fit for one- to five-dimensional models.

Step 3. As a next step, we conducted separate, country specific, unidimensional EFA models for the physical IPV item set and the controlling behaviors item set. Unidimensional EFA models were not assessed for the three-item sexual IPV or psychological IPV item sets, which lack content validity [2] and remain underdeveloped in low- and middle-income country settings [2, 3, 4]. Model fit statistics and item loadings for the unidimensional models of physical IPV and controlling behaviors were adequate for all 36 countries (Tables 3a and 3b and Supplemental Tables S3a and S3b).

Step 4. We, then, performed country-specific confirmatory factor analyses (CFA) and assessed CFA model fit using the same criteria for item loadings and model fit statistics as in the EFA. For the 36 countries, CFA indicated adequate item loadings and model fit for separate, unidimensional models of physical IPV and controlling behaviors (Tables 3a and Table 3b and Supplemental Tables S3a and S3b).

Step 5. We, then, performed separate measurement invariance testing for the seven physical IPV items and the five controlling behavior items. Multiple-group CFA (MGCFA) was performed to test for exact measurement invariance, and alignment optimization (AO) was performed to assess approximate measurement invariance of the unidimensional models (separately) across countries. MGCFA results for the physical IPV items and controlling behavior items demonstrated configural invariance across all 36 countries, but metric and scalar invariance were not achieved (Table 4).

Step 6. Given the absence of metric and scalar invariance in **Step 5**, we applied the AO approach to the seven physical IPV items and the five controlling behavior items separately. Both sets of results are presented in the main paper and suggested approximate measurement invariance across the 36 countries included in the analysis.

References

1. Hu, L. and P.M. Bentler, *Cutoff criteria for fit indexes in covariance structure analysis: Conventional criteria versus new alternatives. Structural Equation Modeling: A Multidisciplinary Journal*, 1999. 6(1): p. 1-55.

2. Costa, D. and H. Barros, *Instruments to assess intimate partner violence: A scoping review of the literature*. Violence and victims, 2016. 31(4): p. 591.
3. Martín-Fernández, M., et al. *Psychological intimate partner violence against women in the European Union: a cross-national invariance study*. BMC Public Health, 2019 Dec;19(1): p. 1-11.
4. Follingstad, D.R. and M.J.J.S.R. Rogers, *Validity concerns in the measurement of women's and men's report of intimate partner violence*. 2013. 69(3-4): p. 149-167.

DETAILED ANALYTICAL STEPS AND FINDINGS. RESULTS AVAILABLE UPON REQUEST

Exploratory factor analyses (EFA) and confirmatory factor analyses (CFA)

1. EFA of all 18 life-time intimate partner violence (IPV) items included in the DHS Domestic Violence module (Table S2), completed using pooled data from 36 countries. One- to five-factor models were specified.

a. Model fit: RMSEA (<0.08) indicated for 1-factor model, while CFI and TLI indicated fit (CFI, TLI ≥0.95) for the 4- and 5-factor models.

b. Loadings: Number of significant loadings varied across models, as did number of loadings ≥0.35.

c. Results summarized in Table 1 below.

2. EFA of all 18 lifetime IPV items, completed individually for each of 36 countries. One- to five-factor models were specified.

a. Theory guided conceptualization of a 4-factor model, with factors theorized to represent different domains of IPV (physical, sexual, psychological, and controlling behaviors).

b. 4-factor model fit: Across all 36 countries, RMSEA ranged from 0.000 to 0.035, CFI from 0.989 to 1.000, TLI from 0.981 to 1.001.

c. Loadings: **Table 2** in this supplemental file summarizes item cross-loading and non-loading (i.e., loading <0.35) across all 36 countries. In 13 countries, emotional IPV items cross-loaded with the physical IPV factor. Additionally, physical IPV items cross-loaded with the sexual IPV factor in up to 7 countries. There was little cross-loading between controlling behavior items and other forms of IPV.

d. Option 1: Limit the number of countries. For 22 countries, a four-factor model showed minimum cross-loadings and four distinct domains of IPV, as theorized.

e. Option 2: Limit the number of items to lifetime physical IPV, sexual IPV, and controlling behaviors (15 items). Estimate one-to-five factor EFAs for each of 36 countries separately. 32 of 36 countries had adequate model fit; however, only 18 countries had no cross-loadings.

3. Based on 2d and 2e results, the team estimated CFAs of four-factor models of 18 lifetime IPV items, individually for each of the 22 countries identified in the EFA (step 2.d.). Thresholds, standardized factor loadings, and modification indices (MIs) were assessed for each item across 22 countries.

a. All estimated factor loadings exceeded 0.35 for all countries.

b. Fit statistics except the chi-square difference test suggested reasonable model fit.

c. However, six countries had very high MIs, six items had very high MIs, and the team decided to maximize inferences about cross-country comparability for more limited items sets across the largest number of countries for 2012-2018.

d. Results are available on request.

4. Factor analyses of lifetime physical IPV items (7 items)

a. EFA of all lifetime physical IPV items (7 items), completed separately for each of 36 countries. One- to two-factor models were specified. Results of these analyses suggested adequate fit of a one-dimensional model.

b. CFA of all lifetime physical IPV items (7 items), completed separately for each of 36 countries. One-factor models were specified using the weighted least square mean and variance estimator. Results of these analyses suggested adequate fit of a one-dimensional model.

5. Factor analyses of controlling behaviors (5 items)

a. EFA for controlling behaviors (5 items) for 36 countries. Results of these analyses suggested adequate fit of a one-dimensional model.

b. CFA for controlling behaviors (5 items) for 36 countries. Results of these analyses suggested adequate fit of a one-dimensional model.

Multiple-group confirmatory factor analysis (MGCFA)

1. WLSMV for 18 items for 22 countries: configural invariance (RMSEA 0.022, CFI 0.989, TLI 0.987), scalar invariance not established (pattern loadings not same)

2. MGCFA Using Maximum likelihood estimation for 36 countries

a. Completed for lifetime physical, sexual, and psychological IPV and controlling behaviors (18 items) for 22 countries: significant differences in configural and metric models, as in metric and scalar models; metric invariance not achieved

b. Completed for lifetime physical and sexual IPV (10 items) for 36 countries: did not demonstrate configural invariance

c. Completed for lifetime physical IPV and controlling behaviors (12 items) for 36 countries: demonstrated configural invariance

d. Completed for lifetime physical IPV (7 items) for 36 countries: demonstrated configural invariance

e. Completed for controlling behaviors (5 items) for 36 countries: demonstrated configural invariance.

Alignment Optimization

1. Alignment optimization for seven physical IPV items

a. Estimated country-specific factor means and variances for the seven lifetime physical IPV items by an optimization process that minimizes the total amount of measurement non-invariance.

b. Assessed the fit of the model and compared the total percentage of non-invariant parameters against the 25% benchmark for obtaining reliable alignment results. The results showed that 12.3% of all parameter estimates were measurement non-invariant.

c. Performed a simulation that focuses on bias and coverage of each parameter. The simulation did not run.

d. Ranked the means of the countries and reported whether they significantly differed from one another

2. Alignment Optimization for five controlling behavior items

a. Estimated country-specific factor means and variances for the five lifetime controlling behaviors items by an optimization process that minimizes the total amount of measurement non-invariance.

b. Assessed the fit of the model and compared the total percentage of non-invariant parameters against the 25% benchmark for obtaining reliable alignment results. The results showed that showed that 6.7% of all parameter estimates were measurement non-invariant.

c. Performed a simulation that focuses on bias and coverage of each parameter.

d. Ranked the means of the countries and reported whether they significantly differed from one another

Note: An AO model with the seven physical IPV items and five controlling behaviour items did not converge.

Table S1. Items included in the World Bank's Women, Business and the Law Index (Women, Business, and Law Data for 2016)

		Mobility					Workplace			Pay			
**Country**	WBL Index	Apply for passport	Travel outside country	Travel outside her home	Choose where to live	Get a job	Law prohibits gender discrimination in employment	Legislation on sexual harassment in employment	Criminal penalties or civil remedies for workplace sexual harassment	Equal renumeration for work of equal value	Same night hours	Work in jobs deemed dangerous	Work in the same industries as men
**Central Asia**													
Kyrgyz Republic	76.9	1	1	1	1	1	1	1	1	0	1	0	0
Tajikistan	75.6	1	1	1	1	1	1	0	0	1	0	0	0
Haiti	61.3	1	1	1	1	1	1	1	1	1	1	1	1
Armenia	80.0	1	1	1	1	1	1	0	0	0	1	1	1
Egypt	45.0	0	1	0	1	0	1	1	1	0	0	0	0
Afghanistan	28.8	0	1	0	0	1	0	0	0	0	0	0	0
Cambodia	75.0	1	1	1	1	1	1	1	1	0	1	1	1
India	68.8	1	1	1	1	1	1	1	1	0	0	0	0
Maldives	70.6	1	1	1	1	1	1	1	1	0	1	1	1
Myanmar	58.8	0	1	1	1	1	0	0	0	0	1	1	0
Nepal	55.6	1	1	1	1	1	0	1	1	0	0	1	1
Pakistan	46.9	0	1	1	1	1	0	1	1	0	0	1	0
Philippines	78.8	0	1	1	1	1	1	1	1	1	1	1	1
Timor-Leste	77.5	1	1	1	1	1	1	1	0	0	1	1	1
Angola	67.5	1	1	1	1	1	1	0	0	1	1	0	0
Benin	74.4	0	1	1	0	1	1	1	1	1	1	0	0
Burundi	73.1	1	1	1	1	1	1	1	1	0	1	1	1
Chad	57.5	1	1	1	0	0	1	0	0	1	1	0	0
Comoros	58.8	1	1	1	0	0	1	1	1	1	1	1	1
DRC	42.5	1	1	1	0	0	0	1	1	0	1	0	0
Ethiopia	71.9	1	1	1	1	1	1	1	1	0	1	0	0
Gabon	51.3	0	1	1	0	0	1	0	0	0	1	0	0
Gambia	74.4	1	1	1	1	1	1	0	0	0	1	1	1
Kenya	78.1	1	1	1	1	1	1	1	1	1	1	1	1
Malawi	80.6	0	1	1	0	1	1	1	1	1	1	1	1
Mali	54.4	0	1	1	0	1	0	0	0	0	1	0	0
Mozambique	76.9	1	1	1	1	1	1	1	1	0	1	0	1
Namibia	86.3	0	1	1	1	1	1	1	1	1	1	1	1
Nigeria	63.1	0	1	1	0	1	0	1	1	0	1	1	0
Rwanda	72.5	1	1	1	0	1	1	1	1	0	1	1	1
Sierra Leone	63.1	1	1	1	1	1	0	0	0	0	1	1	0
Tanzania	84.4	1	1	1	1	1	1	1	1	1	1	1	1
Togo	84.4	1	1	1	1	1	1	1	1	1	1	1	1
Uganda	70.0	0	1	1	0	1	1	1	1	1	1	1	1
Zambia	63.1	0	1	1	0	1	1	0	0	0	1	1	1
Zimbabwe	86.9	1	1	1	1	1	1	1	1	0	1	1	1

Marriage					Parenthood					Entrepreneurship			
No legal provision to obey husband	Woman can be head of household or family	Domestic violence legislation	Obtain divorce	Right to remarry	At least 14 weeks paid leave	Government administers 100% of maternity leave benefits	Paid paternal leave	Paid parental leave	Dismissal of pregant workers prohibited	Sign contract	Register business	Open bank account	Prohibit gender discrimination in access to credit
1	1	1	1	1	1	0	0	0	1	1	1	1	1
1	1	1	1	1	1	1	0	1	1	1	1	1	1
1	1	0	1	1	0	0	1	0	1	1	0	1	0
1	1	0	1	1	1	1	0	1	1	1	1	1	0
0	0	0	0	0	0	0	0	0	1	1	1	1	0
0	1	0	0	0	0	0	1	0	0	1	1	1	0
1	1	1	1	0	0	0	0	0	1	1	1	1	1
1	1	1	1	1	0	0	0	0	1	1	1	1	0
1	1	1	0	0	0	0	1	0	1	1	1	1	0
1	1	0	1	1	1	1	1	0	0	1	1	1	0
1	1	1	0	1	0	0	0	0	0	1	1	1	0
1	1	1	0	0	0	0	0	0	0	1	0	1	0
1	1	1	0	0	0	1	1	0	1	1	1	1	1
1	1	1	1	0	0	0	1	0	1	1	1	1	0
1	1	1	1	1	0	1	0	0	1	1	1	1	0
1	1	1	1	0	1	0	1	0	1	1	1	1	0
1	0	1	1	0	0	0	1	0	1	1	1	1	0
1	0	0	1	0	1	0	1	0	1	1	1	0	0
1	0	1	0	0	1	0	0	0	1	1	1	1	0
0	0	0	1	0	1	0	1	0	1	0	0	0	0
1	1	1	1	0	0	0	0	0	1	1	1	1	0
0	0	0	1	0	1	1	1	0	1	1	1	0	0
1	1	1	1	1	1	0	1	0	1	1	1	1	0
1	1	0	1	1	0	0	1	0	1	1	0	1	0
1	1	1	1	1	0	0	0	0	1	1	1	1	0
0	0	0	1	0	1	1	1	0	0	1	1	1	0
1	1	1	1	0	0	1	1	0	1	1	1	1	0
1	1	1	1	1	0	1	0	0	1	1	1	1	0
1	1	1	1	1	0	0	0	0	0	1	1	1	0
1	0	1	1	0	0	0	1	0	0	1	1	1	0
1	1	1	1	1	0	0	0	0	0	1	1	1	0
1	1	0	1	1	0	1	1	0	1	1	1	1	0
1	1	0	1	0	1	0	1	0	1	1	1	1	0
1	1	1	0	1	0	0	1	0	1	1	1	1	0
1	1	1	0	1	0	0	0	0	1	1	1	1	0
1	1	1	0	1	1	0	0	0	1	1	1	1	1

Assets					Pension			
Equal property ownership	Equal inheritance rights (sons and daughters)	Equal spousal inheritance rights	Equal administrative authority over assets	Valuation of nonmonetary contributions	Full pension benefits	Partial pension benefits	Mandatory retirement age	Periods of absence due to child care accounted for in pension
1	1	1	1	1	0	0	1	1
1	1	1	1	1	0	0	1	1
1	1	0	1	1	1	1	1	0
1	1	1	1	1	1	1	1	1
1	0	0	1	0	1	1	1	1
1	0	0	1	0	0	0	1	0
1	1	1	1	1	0	0	1	0
1	1	1	1	0	1	1	1	0
1	0	0	1	0	1	1	1	0
1	1	1	1	0	0	0	1	0
1	0	0	1	0	0	0	1	0
1	0	0	1	0	0	0	1	1
0	1	1	0	1	1	1	1	0
1	1	1	1	1	1	1	1	0
1	1	1	1	1	0	1	0	0
1	1	1	1	0	1	1	1	1
1	0	0	1	1	1	1	1	0
0	1	1	0	1	1	1	1	1
1	0	0	1	0	0	0	1	0
0	1	1	0	1	0	1	1	0
1	1	1	1	1	1	1	1	0
0	1	1	0	1	1	1	1	1
1	0	0	1	1	1	1	1	0
1	1	0	1	1	1	1	1	0
1	1	1	1	1	1	1	1	1
1	1	1	1	0	1	1	1	1
1	1	1	1	1	0	1	0	1
1	1	1	1	1	1	1	1	1
1	1	1	1	0	1	1	1	0
1	1	1	1	1	1	1	1	0
1	1	1	1	0	1	1	1	0
1	0	0	1	1	1	1	1	1
1	1	1	1	0	1	1	1	1
1	0	0	1	0	1	1	1	0
1	1	1	1	0	1	1	1	0
1	1	1	1	1	1	1	1	1

Notes. DRC=Democratic Republic of Congo; Figures for the GINI are for the years 2009-2018.

[a]Subnational.

Table S2. Items included in the DHS Domestic Violence module

Supplemental Table 2. Items included in the DHS Domestic Violence module	
**Controlling behaviors**	
First, I am going to ask you about some situations which happen to some women. Please tell me if these apply to your relationship with your (last) (husband/partner)?	
(Response options: Yes, No, Don't Know)	
a	He (is/was) jealous or angry if you (talk/talked) to other men?
b	He frequently (accuses/accused) you of being unfaithful?
c	He (does/did) not permit you to meet your female friends?
d	He (tries/tried) to limit your contact with your family?
e	He (insists/insisted) on knowing where you (are/were) at all times?
**Emotional IPV**	
Now I need to ask some more questions about your relationship with your (last) (husband/partner).	
(Response options: Yes, No)	
a	say or do something to humiliate you in front of others?
b	threaten to hurt or harm you or someone you care about?
c	insult you or make you feel bad about yourself?
**Physical IPV**	
Did your (last) (husband/partner) ever do any of the following things to you:	
(Response options: Yes, No)	
a	Push you, shake you, or throw something at you?
b	Slap you?
c	Twist your arm or pull your hair?
d	Punch with his fist or with something that could hurt you?
e	Kick you, drag you, or beat you up?
f	Try to choke you or burn you on purpose?
g	Threaten to attack you with a knife, gun or other weapon?
**Sexual IPV**	
Did your (last) (husband/partner) ever do any of the following things to you:	
(Response options: Yes, No)	
h	Physically force you to have sexual intercourse with him even when you did not want to?
i	Physically force you to perform any other sexual acts you did not want to?
j	Force you with threats or any other way to perform sexual acts that you did not want to do?

Table S3a. Item loadings from exploratory and confirmatory factor analyses of physical intimate partner violence, N=36 Demographic and Health Surveys across 36 countries (2012-2018)

	Country-Specific EFAs (N=36)							Country-Specific CFAs (N=36)						
**Country**	Push you, shake you, or throw something at you? *(0=No, 1=Yes)*	Slap you? *(0=No, 1=Yes)*	Punch with his fist or with something that could hurt you? *(0=No, 1=Yes)*	Kick you, drag you, or beat you up? *(0=No, 1=Yes)*	Try to choke you or burn you on purpose? *(0=No, 1=Yes)*	Threaten to attack you with a knife, gun or other weapon? *(0=No, 1=Yes)*	Twist your arm or pull your hair? *(0=No, 1=Yes)*	Push you, shake you, or throw something at you? *(0=No, 1=Yes)*	Slap you? *(0=No, 1=Yes)*	Punch with his fist or with something that could hurt you? *(0=No, 1=Yes)*	Kick you, drag you, or beat you up? *(0=No, 1=Yes)*	Try to choke you or burn you on purpose? *(0=No, 1=Yes)*	Threaten to attack you with a knife, gun or other weapon? *(0=No, 1=Yes)*	Twist your arm or pull your hair? *(0=No, 1=Yes)*
**Central Asia**														
Kyrgyz Republic	0.95	0.93	0.92	0.91	0.95	0.84	0.92	0.95	0.91	0.90	0.93	0.94	0.96	0.92
Tajikistan	0.80	0.92	0.95	0.89	0.92	0.65	0.92	0.80	0.86	0.93	0.92	1.02	0.74	0.96
**Latin America, Caribbean**														
Haiti	0.92	0.93	0.97	0.95	0.70	0.71	0.89	0.93	0.91	0.94	0.92	0.88	0.67	0.87
**N Africa, W Asia, Europe**														
Armenia	0.96	0.98	0.96	0.97	0.98	0.98	0.95	0.99	0.95	0.98	0.99	0.97	1.00	0.95
Egypt	0.92	0.97	0.90	0.92	0.86	0.83	0.95	0.95	0.96	0.92	0.92	0.90	0.87	0.92
**S, SE Asia**														
Afghanistan	0.95	0.95	0.96	0.95	0.89	0.90	0.93	0.95	0.97	0.96	0.93	0.89	0.86	0.92
Cambodia	0.94	0.95	0.92	0.91	0.60	0.80	0.88	0.96	0.96	0.94	0.96	0.87	0.82	0.92
India	0.89	0.91	0.91	0.94	0.86	0.78	0.93	0.89	0.90	0.93	0.92	0.83	0.81	0.92
Maldives	0.95	0.96	0.98	0.98	0.97	0.94	0.96	0.96	0.94	0.99	0.95	0.83	0.73	0.91
Myanmar	0.87	0.96	0.95	0.97	0.81	0.78	0.92	0.91	0.93	0.97	0.91	0.78	0.85	0.93
Nepal	0.89	0.93	0.94	0.96	0.89	0.84	0.97	0.92	0.97	0.97	0.98	0.96	0.84	0.96
Pakistan	0.97	0.96	0.89	0.92	0.86	0.87	0.95	0.95	0.94	0.85	0.98	0.98	0.87	0.95
Philippines	0.91	0.91	0.94	0.97	0.91	0.89	0.91	0.91	0.86	0.89	0.95	0.93	0.82	0.92
Timor-Leste	0.70	0.83	0.86	0.91	0.68	0.66	0.92	0.76	0.93	0.89	0.87	0.66	0.69	0.87
**Sub-Saharan Africa**														
Angola	0.87	0.92	0.92	0.93	0.82	0.81	0.87	0.84	0.91	0.94	0.92	0.82	0.78	0.88
Benin	0.91	0.87	0.94	0.97	0.90	0.81	0.94	0.89	0.88	0.93	0.93	0.92	0.93	0.91
Burundi	0.89	0.87	0.92	0.93	0.87	0.84	0.90	0.89	0.89	0.92	0.93	0.88	0.76	0.89
Chad	0.92	0.95	0.92	0.92	0.92	0.85	0.94	0.89	0.94	0.94	0.94	0.92	0.82	0.92
Comoros	0.90	0.94	0.92	0.99	0.88	0.93	0.74	0.90	0.93	0.97	1.01	0.82	0.92	0.94
DRC	0.80	0.88	0.87	0.83	0.87	0.85	0.81	0.84	0.85	0.85	0.85	0.91	0.82	0.75
Ethiopia	0.91	0.91	0.90	0.92	0.89	0.76	0.90	0.86	0.95	0.95	0.93	0.80	0.90	0.89
Gabon	0.84	0.80	0.95	0.94	0.84	0.64	0.93	0.85	0.88	0.98	0.94	0.90	0.86	0.91
Gambia	0.78	0.93	0.87	0.81	0.57	0.96	0.86	0.83	0.90	0.84	0.85	0.85	1.07	0.94
Kenya	0.87	0.92	0.94	0.91	0.85	0.79	0.85	0.90	0.89	0.93	0.93	0.85	0.82	0.88
Malawi	0.86	0.94	0.90	0.90	0.83	0.84	0.89	0.89	0.90	0.92	0.94	0.84	0.83	0.89
Mali	0.79	0.83	0.83	0.88	0.79	0.58	0.91	0.77	0.84	0.85	0.86	0.81	0.58	0.74
Mozambique	0.92	0.91	0.94	0.94	0.89	0.83	0.95	0.88	0.96	0.89	0.91	0.84	0.74	0.93
Namibia	0.93	0.94	0.96	0.98	0.87	0.92	0.91	0.90	0.97	0.98	0.93	0.90	0.86	0.89
Nigeria	0.92	0.96	0.92	0.93	0.88	0.78	0.89	0.93	0.96	0.93	0.93	0.83	0.74	0.86
Rwanda	0.95	0.93	0.94	0.94	0.92	0.83	0.89	0.91	0.93	0.95	0.95	0.89	0.84	0.86
Sierra Leone	0.83	0.95	0.85	0.74	0.82	0.83	0.89	0.87	0.95	0.83	0.74	0.76	0.76	0.88
Tanzania	0.87	0.93	0.89	0.92	0.76	0.76	0.81	0.88	0.93	0.93	0.92	0.80	0.80	0.83
Togo	0.91	0.95	0.92	0.91	0.89	0.82	0.89	0.92	0.95	0.91	0.90	0.86	0.89	0.85
Uganda	0.83	0.93	0.90	0.90	0.85	0.74	0.84	0.85	0.94	0.90	0.91	0.85	0.75	0.87
Zambia	0.86	0.90	0.89	0.91	0.87	0.88	0.88	0.86	0.87	0.86	0.93	0.85	0.79	0.90
Zimbabwe	0.88	0.81	0.92	0.93	0.83	0.77	0.88	0.86	0.83	0.90	0.94	0.81	0.79	0.85
Min	0.70	0.80	0.83	0.74	0.57	0.58	0.74	0.76	0.83	0.83	0.74	0.66	0.58	0.74
Max	0.97	0.98	0.98	0.99	0.98	0.98	0.97	0.99	0.97	0.99	1.01	1.02	1.07	0.96

Table S3b. Item loadings from exploratory and confirmatory factor analyses of controlling behaviour, N=36 Demographic and Health Surveys across 36 countries (2012-2018)

Country-Specific EFAs (N=36)						Country-Specific CFAs (N=36)				
**Country**	Jealous or angry if you talk/talked to other men? *(0=No, 1=Yes)*	Frequently accuses/accused you of being unfaithful? *(0=No, 1=Yes)*	Does/did not permit you to meet your female friends? *(0=No, 1=Yes)*	Tries/tried to limit your contact with your family? *(0=No, 1=Yes)*	Insists/insisted on knowing where you are/were at all times? *(0=No, 1=Yes)*	Jealous or angry if you talk/talked to other men? *(0=No, 1=Yes)*	Frequently accuses/accused you of being unfaithful? *(0=No, 1=Yes)*	Does/did not permit you to meet your female friends? *(0=No, 1=Yes)*	Tries/tried to limit your contact with your family? *(0=No, 1=Yes)*	Insists/insisted on knowing where you are/were at all times? *(0=No, 1=Yes)*
**Central Asia**										
Kyrgyz Republic	0.78	0.62	0.97	0.91	0.74	0.89	0.63	0.79	0.99	0.68
Tajikistan	0.75	0.75	0.83	0.84	0.68	0.73	0.73	0.87	0.90	0.65
**Latin America, Caribbean**										
Haiti	0.85	0.84	0.87	0.91	0.74	0.85	0.80	0.89	0.78	0.76
**N Africa, W Asia, Europe**										
Armenia	0.81	0.77	0.85	0.92	0.81	0.77	0.80	0.86	0.81	0.79
Egypt	0.55	0.75	0.75	0.79	0.70	0.41	0.65	0.74	0.68	0.68
**S, SE Asia**										
Afghanistan	0.79	0.85	0.77	0.66	0.67	0.74	0.84	0.78	0.76	0.71
Cambodia	0.95	0.93	0.77	0.88	0.92	0.91	0.94	0.82	0.93	0.90
India	0.74	0.85	0.72	0.85	0.74	0.74	0.86	0.71	0.82	0.74
Maldives	0.77	0.84	0.92	0.77	0.83	0.82	0.95	0.93	0.70	0.67
Myanmar	0.81	0.91	0.84	0.80	0.73	0.80	0.89	0.87	0.91	0.69
Nepal	0.86	0.87	0.76	0.89	0.75	0.89	0.88	0.77	0.86	0.72
Pakistan	0.82	0.79	0.90	0.85	0.88	0.82	0.85	0.95	0.88	0.80
Philippines	0.87	0.90	0.82	0.82	0.77	0.84	0.89	0.84	0.80	0.79
Timor-Leste	0.80	0.72	0.83	0.92	0.73	0.75	0.66	0.90	0.83	0.73
**Sub-Saharan Africa**										
Angola	0.85	0.86	0.81	0.82	0.81	0.82	0.78	0.87	0.76	0.85
Benin	0.83	0.84	0.79	0.84	0.74	0.80	0.78	0.83	0.81	0.76
Burundi	0.81	0.86	0.89	0.86	0.91	0.87	0.87	0.89	0.87	0.90
Chad	0.87	0.88	0.87	0.86	0.70	0.84	0.87	0.90	0.88	0.76
Comoros	0.87	0.83	0.81	0.77	0.83	0.83	0.75	0.87	0.90	0.82
DRC	0.80	0.74	0.77	0.70	0.72	0.79	0.71	0.77	0.71	0.71
Ethiopia	0.53	0.87	0.85	0.75	0.78	0.50	0.82	0.85	0.79	0.78
Gabon	0.84	0.86	0.68	0.60	0.79	0.82	0.88	0.85	0.66	0.71
Gambia	0.63	0.82	0.77	0.95	0.78	0.59	0.76	0.90	0.92	0.75
Kenya	0.86	0.76	0.85	0.83	0.79	0.79	0.82	0.85	0.81	0.78
Malawi	0.81	0.84	0.78	0.80	0.71	0.77	0.77	0.84	0.82	0.70
Mali	0.77	0.84	0.83	0.85	0.79	0.85	0.82	0.83	0.88	0.74
Mozambique	0.91	0.85	0.86	0.91	0.83	0.89	0.82	0.92	0.85	0.86
Namibia	0.93	0.86	0.82	0.81	0.83	0.90	0.95	0.83	0.86	0.82
Nigeria	0.63	0.71	0.87	0.87	0.71	0.67	0.71	0.89	0.87	0.73
Rwanda	0.90	0.84	0.92	0.88	0.85	0.87	0.83	0.85	0.78	0.83
Sierra Leone	0.91	0.89	0.78	0.88	0.63	0.85	0.85	0.81	0.70	0.72
Tanzania	0.81	0.77	0.83	0.76	0.72	0.81	0.77	0.81	0.85	0.74
Togo	0.84	0.79	0.85	0.82	0.76	0.79	0.71	0.81	0.85	0.78
Uganda	0.84	0.78	0.87	0.81	0.79	0.82	0.76	0.81	0.78	0.79
Zambia	0.87	0.83	0.79	0.78	0.72	0.89	0.82	0.78	0.76	0.73
Zimbabwe	0.80	0.79	0.89	0.82	0.76	0.74	0.79	0.93	0.77	0.83
Min	0.53	0.62	0.68	0.60	0.63	0.41	0.63	0.71	0.66	0.65
Max	0.95	0.93	0.97	0.95	0.92	0.91	0.95	0.95	0.99	0.90

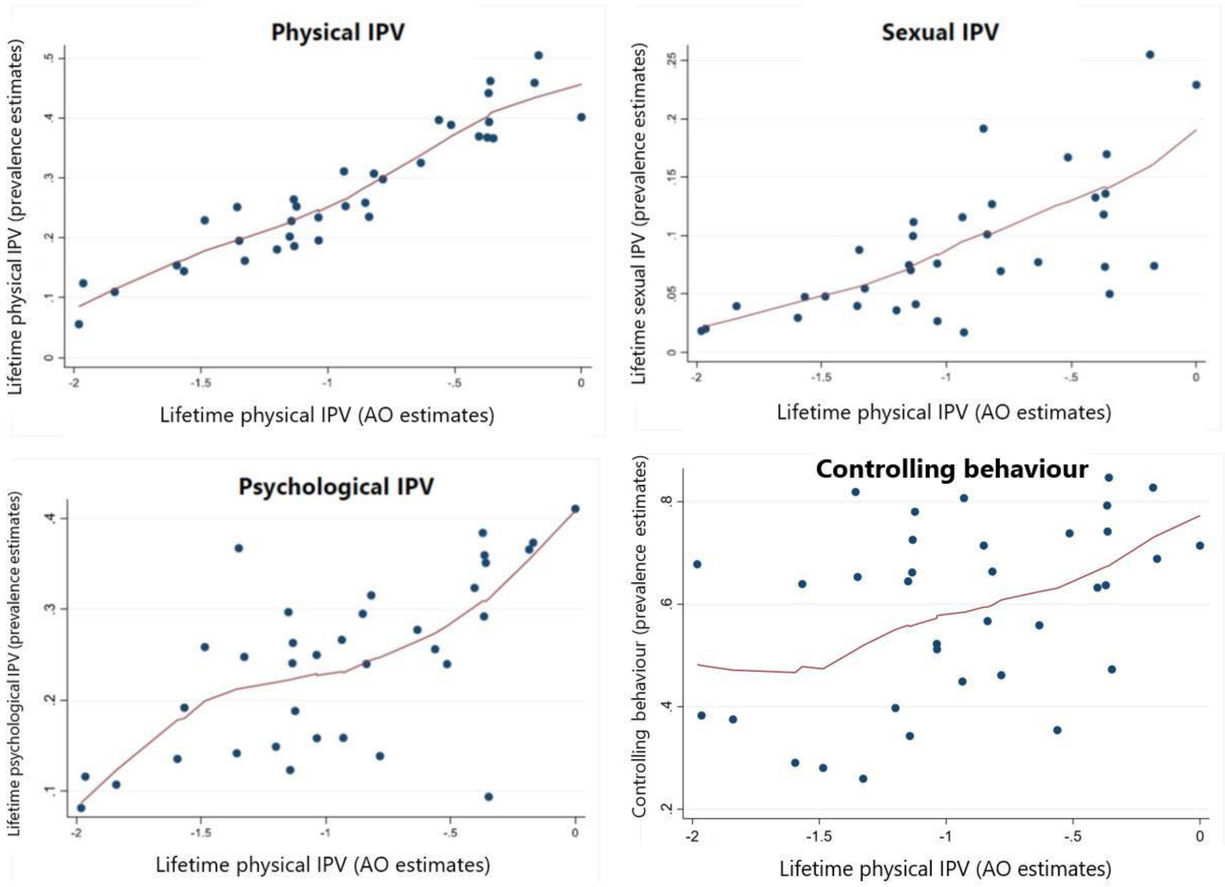

Figure S1. Correlations of prevalence estimates and alignment optimization estimates of lifetime intimate partner violence across 35 countries (outlier removed), 2012-2018.

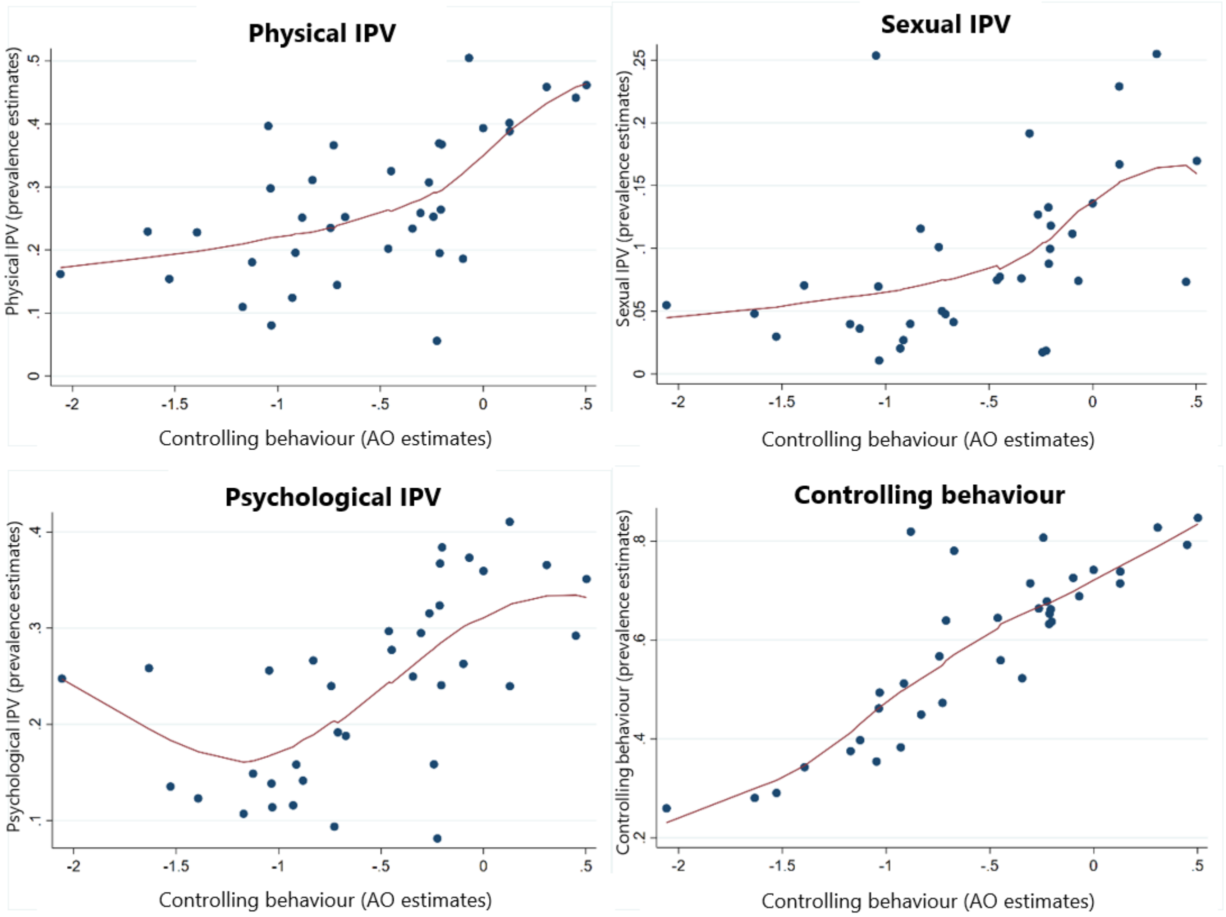

Figure S2. Correlations of prevalence estimates and alignment optimization estimates of controlling behaviour across 36 countries, 2012-2018.
